# The Impact of Transformational Leadership on Organizational Commitment and Intention to Stay: Empirical Evidence From China’s Performing Arts Industry

**DOI:** 10.3389/fpsyg.2022.874803

**Published:** 2022-05-06

**Authors:** Hengzhe Xu, Zhong Wang, Naiyu Lian, Asif Khan, Lei Zhang

**Affiliations:** ^1^Department of Arts Management, Xinghai Conservatory of Music, Guangzhou, China; ^2^Faculty of Humanities and Social Sciences, City University of Macau, Macau, Macao SAR, China; ^3^Department of Marketing and Distribution Management, College of Management, National Kaohsiung University of Science and Technology, Kaohsiung City, Taiwan; ^4^Department of Music, Guangdong Polytechnic Normal University, Guangzhou, China

**Keywords:** transformational leadership, person–job fit, social capital, psychological capital, organizational commitment, performing arts organizations

## Abstract

As part of the cultural industries, performing arts has been playing an important role in enriching people’s spiritual life, leveling culture and education, creating jobs, and even making economic benefits. Hence, a significant methodology is required to tackle the complicated concepts of transformational leadership (TL) and social factors in an arts industry context. This article aims to observe the direct impacts of TL on organizational commitment (OC). Furthermore, it examined the indirect effects of TL on OC and intention to stay (ITS) *via* person–job fit (PJF), social capital (SC), and psychological capital (PC). According to the results of this research, TL was discovered to have a positive impact on OC and PJF while having no significant effect on ITS. Furthermore, PJF had a significant impact on SC. Moreover, SC significantly influenced PC. In addition, PC was discovered to be in a significant correlation with OC while having no significant association with ITS. Finally, OC was also in a significant relationship with ITS.

## Introduction

Despite a considerable contemporary interest in the economic and social roles played by the “cultural” or “creative” industries, less attention has been paid to the livelihood experiences of the cultural workforce underpinning these developments (Coulson, 2010). As part of the cultural industries, performing arts has been playing an important role in enriching people’s spiritual life, leveling culture and education, creating jobs, and even making economic benefits. Numerous psychological and behavioral studies have been performed on transformational leadership (TL); nevertheless, these studies did not focus on performing arts industries. This implies that a significant methodology is required to tackle the complicated concepts of TL and social factors in a performing arts industry context. Globally, performing art groups, as the core content providers in the performing arts industry, have encountered many challenges in recent years, such as aging audiences, high competition in the market, tight funding, and the loss of outstanding players. Besides, performers’ career goals often come at the expense of their own health, such as musicians’ injuries and illnesses (Brodsky, 2006) and opera singers’ professional anxiety (Sandgren, 2009). Music and the arts are often important motivators because the performance itself can bring enjoyment and a sense of accomplishment to the performer. However, a performer’s love and enthusiasm for the performing arts by itself is not enough to promote the desire to remain in the performing community (Brodsky, 2006). It has also been suggested that one way to increase performers’ organizational commitment (OC) is to increase their autonomy as decision-makers (Shadur et al., 1999; Raelin, 2011). However, in fact, performers in performing arts groups are often less likely to be involved in decision-making. For example, in orchestras, the role and influence of the conductor and artistic director are valued more than the concern for one of the violinists. Some performers feel that they are not involved in decision-making and that their opinions and suggestions are not taken seriously (Oakland and Ginsborg, 2014). In contrast to traditional leadership, TL focuses on the needs of the organization’s members, inspires, empowers, and helps subordinates to recognize and understand the value of their work, and develops subordinates to become better decision-makers in order to improve organizational performance and achieve organizational goals. Therefore, it is valuable to evaluate the impact of TL on performers’ OC and intention in a special organization, such as performing art groups.

As a sort of implied rule conformity, societal norms intelligently affect the assessment of leadership and creativity of social people (York and Lenox, 2014). Earlier findings have described creativity as a method by which an individual provides original and applied notions, concentrating on the effect of inherent individualities of the topic of creative concepts on creativity, for instance, an individual’s character, thinking methodology, information storage, and inspiration (Kandler et al., 2016). Nevertheless, only some findings have investigated the impact of creativity from a societal viewpoint, such as the leadership style, disregarding the point that creativity is similarly a social development topic to societal perception and belief (Du et al., 2021). The leadership approach can indicate the efficiency of the company. This is an essential characteristic that affects the advancement of the employees and the organization’s operation (Manzoor et al., 2019). TL is deemed as an important leadership approach and it is described as a leadership method that allows the leader to engage with groups to recognize the change, furthermore developing a vision to inspire and implement the change along with the loyal individuals of the organization (Bushra et al., 2011). TL has developed the interest among the investigators as a result of its substantial and optimistic effects on managerial and employees’ performance (Manzoor et al., 2019).

Individuals’ commitment to their organizations has been considered a critical issue in management studies (Rego et al., 2016). In addition, the employees’ sense of belongingness is a crucial element for an organization, and it is impacted by leadership. Hence, scholars investigating the leadership take into account the essential concept of OC, which can be described as allegiances to the beliefs and objectives of the company, dependence, and ethical obligation to stay in their company (Ibrahim Alzamel et al., 2020). Various findings draw attention to the impact of leadership on OC. It is thus essential to explore leadership as a means of discovering the preferred managerial framework of optimism (Rego et al., 2016). Hence, some companies incorporate assessment and execution of different kinds of leadership in their organizational strategy. Consequently, some scholars have discovered the importance of TL in enhancing OC (Walumbwa et al., 2008). Transformational leaders know the demands of corporations and people who follow the leaders as their ideals of charisma, honesty, and truthfulness. Furthermore, transformational leaders direct their people toward their lives and their jobs (Gardner et al., 2005). TL is distinguished by being clear, honorable, and honest with everyone, working in agreement with their beliefs, principles, and ideas, and providing a genuine and authentic association (Walumbwa et al., 2008). This implies that the greater the degree of apparent leadership, the more the dedicated people are toward accomplishing their goals (Kernis and Goldman, 2005). TL is in a significant relationship with OC because of the developmental methodology of the transformational leader, as it can significantly impact the actions and opinions of workers, improving OC behaviors and efficiency (Walumbwa et al., 2008; Rego et al., 2012). This triggers followers to believe further dedicated to attaining the aims, based on their level of perceived TL (Kernis and Goldman, 2005; Rego et al., 2016). This research article focuses on measuring the impact of TL on OC in the performing arts industry.

Worker retention is a crucial concern and a huge issue for organizations (Deery and Jago, 2015). Improving worker’s intention to stay (ITS) is an essential objective for organizations because of the great costs related to employing, positioning, and coaching new applicants (Deery and Jago, 2015; Sobaih et al., 2020). ITS is theorized as workers’ ITS with their current organization for a long time (Johari et al., 2012). ITS is found as a crucial element of turnover conduct and is inspired by many workers’ job feelings, involving satisfaction toward their jobs and their OC (Gruman and Saks, 2011; Milliman et al., 2018). ITS is the greatest forecaster of worker retention and issues impacting ITS are expected to impact worker turnover (Brewer et al., 2012). The notable factors of ITS within their corporations are leaders’ skills to inspire, motivate, encourage, and fulfill their workers (Shuck and Herd, 2012). Leadership is a crucial factor of the industry’s potential and future (Ohunakin et al., 2019). To better understand the employee performance conduct in organizations, it is important to examine TL (Dai et al., 2013). TL is frequently linked with employees’ ethical principles (Joo and Nimon, 2014). Hence, this article aims to study the relationship of TL with the ITS.

Person–job fit (PJF) is described as the connection among the skills of an individual and the needs of the workplace, or the requirements, or aspirations of an individual and the actual provisions by the workplace (Edwards, 1991), which relates with the corresponding fit (Boon et al., 2011). Employing fit theory, especially with an emphasis on PJF, this research fulfills the research gap in the current TL studies regarding the knowledge of finding the right TL strategy to influence PJF (Bui et al., 2017). In addition, this research similarly analyses the relationship between PJF and social capital (SC). SC, as the fundamental basis of human resource management (HRM), is usually recognized as a unique method to supervise individuals who pursue to attain competitive benefit as a result of the strategic growth of well dedicated and competent personnel (Raja et al., 2018). PJF can be inferred as a driving force of SC. Investigation on value equivalence has indicated that fulfilled and dedicated workers report greater PJF. Similarly, workers with values matching with other workers are further expected to believe those workers (Tsui and O’reilly, 1989). Once people are employed in a framework described by goal/objective comparison, they are further expected to adopt organizational standards and cooperate with other individuals in a method, which encourages a better amount of bonds, shared trust, and a common attitude, which is the basis of SC (Raja et al., 2018). Moreover, SC was found to be in a significant relationship with psychological capital (PC) (Raja et al., 2018). Hence, this study intends to link SC with PC.

PC is a state of optimistic personal psychological improvement described by four psychological characteristics, including optimism, self-efficacy, resilience, and hope. Self-efficacy can be described as an understanding equal to self-assurance in an individual’s capability to accomplish particular responsibilities. Moreover, optimism is described as the emotional intent and beliefs to assume the finest constructive outcomes that can significantly impact individual’s physical and psychological wellbeing. In addition, hope represents anticipation that offers individuals assumed fortitude and commitment to devote their energies in order to accomplish their aims. Finally, resilience is the capability to adjust in correspondence to confronting difficulties or considerable risks (Luthans and Youssef, 2004; Mahfud et al., 2020). In addition, corresponding to previous literature, PC is found to be in a significant relationship with OC (Peng et al., 2013) and ITS (Karatepe and Karadas, 2014). Consequently, this article analyzes the relationship of PC with OC and ITS. OC is significantly associated with ITS (Ibrahim Alzamel et al., 2020). Hence, this research article also examines the association between OC and ITS. This study also intends to fulfill the following significant research gaps for TL. First, this study intends to explore the direct impacts of TL on OC. Second, this study examines the indirect effects of TL on OC and ITS *via* PJF, SC, and PC.

## Literature Review

### Transformational Leadership

The current leadership theory is usually described as a volatile and mutual method among individuals chasing a shared objective (Komives and Dugan, 2010). In an organizational setting, this concept considers the interactive activities, including the associations between managers and employees. For instance, the distinctive relationship of a leader with an employee accounts for exceptional execution of job activities and achievements for the company. Investigators have numerous opinions regarding the part of leadership and its impact on the attainment and closure of any plan, company, or organization; nonetheless, many of the researchers approve that those leadership methods have a vital part in the development and achievement of any company. Leaders are engaged in recognizing the company’s objectives, encouraging conduct related to these objectives, and controlling the company’s culture (Erkutlu, 2008). Leadership can similarly be described as the capability to inspire, promote, and allow employees to participate related to the success and competencies of the company. The latest literature demonstrates that TL is a well-known leadership method, and it highlights philanthropy and altruïsm for the improvement of the workers or companies (Manzoor et al., 2019). In the research on modern HRM, TL is a subject that has drawn a great level of interest from researchers and specialists (Banks et al., 2016). It is also described as a motivational process through which leaders’ behavior affects the attitudes and behavior of their followers (Hammond et al., 2015). Empirical findings have frequently proved the significance of TL in forecasting employees’ self-efficacy (Walumbwa et al., 2008), optimism (Lu et al., 2018), self-esteem (Matzler et al., 2015), innovation implementation behavior (Michaelis et al., 2010), task performance (Brouer et al., 2016), shared values (Lee et al., 2010), and organizational citizenship behavior (OCB) (Zacher and Jimmieson, 2013). Nevertheless, comprehensive knowledge of TL and its operational methods is still uncertain (Bui et al., 2017).

### Organizational Commitment

Becker (1960) performed the initial investigations of OC developed in the 1960s. According to him, a commitment was a concept that described the different kinds of behaviors deemed by people as an investment in companies that eventually confine their actions and potential. Other researchers have focused on this subject, by creating a structure, however, not a consensual description recognized by everyone. After examining all descriptions, there was somewhat similarity to each one of them and it aided to describe the concept as a psychological connection between an employee and a company, and a power that alleviated and focused their behavior (Meyer and Herscovitch, 2001). Furthermore, corresponding to Rego et al. (2016) and the Meyer and Allen’s multidimensional prototype (1991), comprising affective commitment, normative commitment, and continuance commitment has larger agreement, approval, and utilization. The notion of OC indicates that workers make a choice to leave or stay with the company (Meyer et al., 1993). OC is employed to imply if a worker will decide to maintain his affiliation in the company (Allen and Meyer, 1996). It implies a mental state that defines the association between a worker’s job and the company. Hence, it can be inferred that OC is significant for employees and managerial goals as it operates as a path between the industry and the employees (Patiar and Wang, 2016).

### Intention to Stay

The workforce is an essential component of any organization and, therefore, employees are always considered as the greatest asset of an organization. Most organizations recognize the importance of human resources in realizing the success of their businesses (Johari et al., 2012). ITS is theorized as a person’s intent to continue with their current organization and was found to have the largest total impaction turnover (Price and Mueller, 1981). ITS is found as a significant factor of real turnover conduct and is manipulated by several worker job approaches, involving OC and job satisfaction, which can be associated with work spirituality and commitment (Saks, 2011; Milliman et al., 2018).

### Person–Job Fit

The PJF is described as the association between the employee’s capabilities and the requirements of a position, or the demands of an employee, in comparison with the actual job offering (Edwards, 1991; Boon et al., 2011). PJF implies the connection between work reinforcement structure and the need structure of the employee (Xie et al., 2016). Consequently, PJF is an essential notion for workers and managers. A significant quantity of investigation has expressed significant relationships between PJF and OC (Scroggins, 2008; Atmojo, 2015).

It can be speculated that a greater PJF indicates greater individual creativity, and these workers will make improved usage of their capabilities, hence, promoting the satisfaction of different goals (Edwards, 1991), and with a smaller amount of concern regarding emotional commitment. In contrast, workers with lesser PJF might have difficulties in managing coworkers and cannot take benefit of their strengths; consequently, these workers might pay extra consideration to emotional commitment (Yang et al., 2019). In the employing fit theory, especially with an emphasis on PJF, this research fulfills the gap in the current TL research. Therefore, more investigation regarding the impact of TL on the role workers’ assessments of their jobs and businesses and their associated PJF is deemed to be necessary. It improves the knowledge of the relationship between TL and different results by analyzing PJF as one of the earlier unexplored mediators (Yukl and Mahsud, 2010; Bui et al., 2017).

### Social Capital

The SC is described as a resource that occurs in social interactions between people, groups, and societies to attain shared advantages (Coleman, 1988). As curiosity in SC has increased, several experiments have discovered this idea in organizations (Bolino et al., 2002; Andrews, 2010; Ko, 2021). SC is perhaps an essential social means, rooted in the societal relationships in a company, for enhancing the company’s results by encouraging information transmission and distribution. In addition, with an importance in the organizational setting, these studies have deemed SC as a representative of the whole organization (Kiss et al., 2014; Berthelsen et al., 2016; Ko, 2021). SC is a multidimensional notion that contains bonding SC, which can be described as communications among individuals, bridging SC, which can be explained as collaborations with external individuals, and linking SC, which is described as faith in the power and official organizations (Putnam, 2000; Szreter and Woolcock, 2004). The difference of the abovementioned three kinds of SC indicates that SC is recognized by encouraging and trustful associations between associates and among workers and the organization. As a collective ability of a company, SC contains cooperation, faith, and organizational integrity in an organizational setting (Oksanen et al., 2010; Kiss et al., 2014; Berthelsen et al., 2016). In this study, built on Leana and van Buren (1999) description, SC indicates that mutual benefits indicate the trait of social connections between people in a company. SC was operationalized comprising three dimensions of SC, including structural SC that is related to connectedness, relational SC that is associated with shared trust, and cognitive SC that can be affiliated with shared principles (Nahapiet and Ghoshal, 1998; Ko, 2021).

### Psychological Capital

According to the study by Luthans et al. (2007a), optimistic organizational conduct is centered on the personal level and analyzes different influences and constructive mental abilities, which can be evaluated and assessed. Improvement aims at a viewpoint of positive outcomes in operation that lead to enhanced organizational functioning (Luthans and Youssef, 2004; Luthans et al., 2005). PC is described as an optimistic psychological condition of growth that is related to the individual’s confidence inputting the required efforts to prosper at completing difficult tasks which are also known as self-efficacy. Furthermore, to make an optimistic acknowledgment about being successful is also called optimism. In addition, be diligent in relation to objectives and take the necessary steps to redirect the ways to achievement, for instance, hope and, if disturbed by difficulties, continue and recover, to accomplish, which is known as resilience (Luthans et al., 2007a; Rego et al., 2012).

## Hypothesis Development

### Transformational Leadership and Organizational Commitment

According to Meyer and Allen (1991) framework, OC is a predictor of the conduct of employees in the company, specifying the way these employees place themselves and the way they are linked to the organization. This has an impact on absenteeism and job performance (Mathieu and Zajac, 1990; Ng and Feldman, 2008). Every element of the Meyer and Allen’s OC framework (1991) describes employees’ relationship to the company (Meyer and Allen, 2004). Affective commitment is related to individual traits (Mathieu and Zajac, 1990), for instance, apparent competency, age because as employees’ age increase, they have fewer work prospects, and educational achievement because a negative association exists with this component and it is linked to the likelihood of further alternate work. Nevertheless, a few researchers point to specialized characteristics as antecedents of this aspect, e.g., rank in the company that is linked to organizational dependency (Mathieu and Zajac, 1990), professional knowledge, and satisfaction related to job combined with a better sense of accountability that consequently leads to higher OC (Allen and Meyer, 1996). Individual qualities, the choice of shifting work, and tenure are part of calculative OC. Finally, normative OC is linked to socialization practices from the worker’s association with the company (Rego et al., 2016). TL is significantly linked to improved OC because of the behavioral method of the transformational leader (Walumbwa et al., 2008; Rego et al., 2016) as it can certainly impact the actions and mindsets of workers, creating OC, performance, and organizational citizenship conduct (Rego et al., 2012). These reasons make employees to consider more commitment while accomplishing the organizational objectives and goals, as a result of their level of apparent authenticity (Kernis and Goldman, 2005; Rego et al., 2016). More precisely, TL aids to improve OC and trust. Leaders engaged in TL urge their employees to feel artistic, which could have a significant impact on their employees’ OC (Sobaih et al., 2020). Another study supported this argument by stating TL can improve employees’ OC and motivational degree by becoming engaged to resolve difficulties creatively and knowing their demands (Walumbwa and Lawler, 2003). Meyer et al. (2004) classified OC into three attitudes, namely, affective commitment related to workers’ emotive connection to their companies, normative commitment involved with workers’ perception of responsibility to stay, and continuous commitment linked to workers’ apparent losses, in case they intend to leave the company. Earlier findings related to TL impact on OC have examined only one dimension of OC, for instance, affective commitment (Dai et al., 2013; Sobaih et al., 2020). Therefore, the following hypothesis can be postulated.

H1: TL positively influences OC.

### Transformational Leadership and Intention to Stay

It can be inferred that the leadership method is a trustworthy forecaster of ITS (Chen and Wu, 2017). Factors impacting workers’ ITS are expected to impact their turnover (Brewer et al., 2012). Consequently, the leaders’ skills to motivate, inspire, and fulfill their workers are important forces of workers’ ITS with their companies (Shuck and Herd, 2012). Furthermore, a study examined the way personal and organizational aspects impact worker ITS. TL was one of the independent variables of the research. TL aims to the achievement of duties and wellbeing of employees. The findings revealed that TL is a powerful predictor of ITS (Kim and Jogaratnam, 2010). Furthermore, another study examined the association between leadership ways and ITS. According to the results, TL increased an employee’s ITS (Wells and Peachey, 2011). Moreover, Chen and Wu (2017) have similarly discovered that leadership conducts, particularly TL, impact ITS in the service industry. Workers are anticipated to demonstrate constructive actions, for instance, ITS, whenever they get proper attention from their leaders (Book et al., 2019; Sobaih et al., 2020). Therefore, the following hypothesis can be postulated.

H2: TL positively influences employee ITS.

### Transformational Leadership and Person–Job Fit

TL is described as a leadership method that allows the leader to engage with groups to recognize the change, additionally develop a vision to motivate and employ the change alongside the loyal workers of the organization (Bushra et al., 2011). In contrast, PJF is described as the association between the employee’s capabilities and the requirements of a position, or the demands of an employee, in comparison with the actual job offering (Edwards, 1991; Boon et al., 2011). The relationship between TL and PJF can similarly be described by leaders’ capability to deal with workers’ demands. Whenever workers are deemed separately, they have a tendency to be encouraged to develop additional work abilities (Bui et al., 2017). TL improves workers’ demands from smaller degrees, including functioning and protection to greater degrees, including respect and self-actualization with regard to Maslow’s hierarchy of needs (Albritton, 1998). To be certain, it is suggested that TL methods, for instance, encourage the enthusiasm to affect workers’ feelings toward their careers and improve the value in employment, including connecting the work to a larger purpose (Bui et al., 2017). Similarly, a study by Enwereuzor et al. (2018) reported TL, PJF, and work engagement among hospital nurses. It examined the moderating impact of PJF on the relationship between TL and work engagement. According to the results of this study, TL had a significant impact on work engagement, while PJF was found to significantly moderate the relationship between TL and work engagement. Moreover, it can be inferred that TL might encourage their employees to work ahead of expectations. Such knowledge can nevertheless vary on the amount of the PJF of the employees. The logic is that it would be simpler for workers with an elevated degree of PJF to be further involved with their job while encouraged by TL in comparison with those employees with lower degrees of PJF (Enwereuzor et al., 2018). Furthermore, another study similarly suggested that PJF was assessed as a mediating variable to study the effect of TL. According to the findings of the research, it was discovered that the association between TL and performance was successfully mediated by PJF (Chi and Pan, 2012). Consequently, it is fair to believe that TL enhances workers’ PJF. Established on the mentioned reasons, the following hypothesis can be postulated.

H3. TL positively influences PJF.

### Person–Job Fit and Social Capital

There are several explanations of apparent PJF in research. For this research, PJF can be defined as the equivalence and comparison between employees and their companies (Piasentin and Chapman, 2006). The fit consists of supplementary fit, including the equivalence attained when the individual and the company acquire identical traits, and complementary fit, including the relationship between anticipation and demands of the company and prospects and requirements of employees (Raja et al., 2018). Various findings have underlined that PJF is associated with a variety of consequence variables, including job exploration and assortment, job fulfillment, personal performance, dedication, turnover, and worker welfare (Kim et al., 2005; Biswas and Bhatnagar, 2013). This study focuses on PJF’s correlation to a further company-positioned result variable, SC. It can be contended that PJF is an influential driver of SC. A study on value comparison has indicated that pleased and dedicated workers state greater PJF (Kim et al., 2005; Raja et al., 2018). Similarly, people whose ideals fit with other workers are further prone to trust those workers (Tsui and O’reilly, 1989). Once people are employed in a framework described by importance/objective comparison, they are further expected to assume organizational standards and work together with others who encourage a shared attitude, and shared trust, which constitutes SC. The improvement of SC similarly involves personnel to create a common mutual perspective and to hypothesize positions in a comparable style (Nahapiet and Ghoshal, 1998). Consequently, it is believed that workers share comparable morals with their company, and they are probable not only to recognize the widespread organizational customs, guidelines, and traditions; nevertheless, they will correspondingly interrelate with other personnel in a method that enables joint approaches of planning and understanding actions and circumstances, thus forming SC. In addition, the social identity theory highlights that entities that distribute related beliefs and aims with their company are additionally expected to recognize with their company. Consequently, people are further encouraged to grow to believe and shared viewpoints with others over this method of communicating principles and recognition that promotes the growth of SC. Therefore, the following hypothesis can be postulated.

H4. PJF positively influences SC.

### Social Capital and Psychological Capital

SC has been labeled as the point of selfless inclinations and the degree of shared trust among individuals in a society. SC is established and theorized through societal relationships (Seibert et al., 2001; Mahfud et al., 2020). Consequently, for the objectives of this research, SC is the value achieved by people out of societal relations to improve their societal capability. People with great SC have a tendency to be extra reliable, further collaborative, and less egocentric (Hasan et al., 2017). Some researchers consider SC as a useful source that opens door to many other supplies for instance investment, market knowledge, and consumers. In the framework of the service industry, SC is created over societal relations in the service setting. Earlier findings have defined SC with the qualities of unity, relationships, societal organization, and faith (Paiva et al., 2014; Mahfud et al., 2020). In addition, Chia and Liang (2016) examined the impact of creativity and SC on industrial intents in 213 service sector individuals. The findings discovered that creativity and SC are important considerations in creating business objectives. The function of the theory of planned behavior (TPB) prototype to the development of objectives for societal entrepreneurship asserts that SC could affect societal business intent over perceived behavioral control (PBC) (Zaremohzzabieh et al., 2019). Correspondingly, alteration of the TPB framework by Malebana (2016) acknowledged that PBC mediates SC in entrepreneurship intent. Corresponding to TPB theory, PBC indicates to control attitudes regarding the presence of circumstances that assist or confuse the manner of conduct, and opinions regarding the force of these aspects. The idea of PBC is similar to the PC theory that contains resilience, self-efficacy, hope, and optimism (Mahfud et al., 2020). Therefore, the following hypothesis can be postulated.

H5. SC positively influences PC.

### Psychological Capital and Organizational Commitment

A substantial relationship similarly occurs among PC and OC. Luthans and Jensen determined an extremely powerful constructive association between the PC and the evaluation of the dedication of employees to the objective, principles, and aspirations of the organization (Peng et al., 2013). Furthermore, another research by Larson and Luthans (2006) stated a substantial constructive relationship between PC and job satisfaction and OC. Moreover, research conducted in China investigated the impact of PC on job performance and OC and discovered, which resiliency, hope, and optimism had a constructive impact on worker job performance, organizational citizenship, and OC. Consequently, the PC, of a worker, had a significant influence on OC (Peng et al., 2013). Hence, the following hypothesis can be postulated.

H6. PC positively influences OC.

### Psychological Capital and Intention to Stay

The current research suggests that PC possesses a particular trait resource decreasing absence and turnover intents (Karatepe and Karadas, 2014). Even though inadequate, there are findings to encourage these associations. Particularly, Avey et al. (2006) concluded that PC was a forecaster of unintentional absence. Research revealed a significant correlation between PC and ITS (Avey et al., 2009). Furthermore, a meta-analytic study conducted by Avey et al. (2011) demonstrated PC to significantly impact ITS. Moreover, evidence also exists regarding PC as moderators or antecedents of ITS in the service management studies. PC’s self-efficacy is discovered to increase employees’ ITS (Karatepe and Karadas, 2014). In addition, PC’s hope is considered to reduce turnover and increase employees’ ITS (Yavas et al., 2018). Regardless of such conclusions, there is still a lack of investigation concerning PC, expressed by optimism, hope, resilience, and self-efficacy, on ITS (Karatepe and Karadas, 2014).

The PC has been described as an individual’s capability to deal with difficult situations with patience and endurance and to complete the desired goals to achieve success (Avey et al., 2009). According to the above description of PC, workers possessing high PC will have high capability to deal with challenging work situations and, hence, will have high ITS at their jobs. As individuals make choices regarding performing a task, and persistence to achieve the task with their self-efficacy principles (Bandura, 1982), it is probable that by trusting on their self-efficacy beliefs to manage the situation and varying circumstances (Bandura, 2012), their ITS rises (Aria et al., 2019). A recent study by Aria et al. (2019) explored the mediation impact of PC and organizational support on the association between leadership and ITS. It was found that leadership was in a significant relationship with ITS, perceived organizational support, and PC. Furthermore, PC was also found to be in a significant relationship with ITS. Hence, the following hypothesis can be postulated.

H7. PC positively influences employee ITS.

### Organizational Commitment and Intention to Stay

The OC is described as allegiances to the beliefs and objectives of the company, idea of fitting, dependence, and ethical responsibility to stay in their company. The OC is inspired by job life quality. Once the workers are pleased with their job life, they will be further compelled and remain in their company (Ibrahim Alzamel et al., 2020). Furthermore, another research testified that the person’s job life quality had a significant impact on OC (Ghoddoosi-Nejad et al., 2015). In addition, OC is found to be in a significant relationship with ITS (Lee et al., 2017). This implies that the OC of workers to their company by fulfillment and acknowledging job setting circumstances will reduce turnover intent and increase employees’ ITS. Consequently, OC is an essential forecaster of ITS; workers who demonstrated great OC and connection to their company were less expected to have plans to leave and devote long periods in the same company (Mothoa, 2016; Ibrahim Alzamel et al., 2020). Hence, the following hypothesis can be postulated.

H8. OC positively influences employee’s ITS.

**FIGURE 1:**
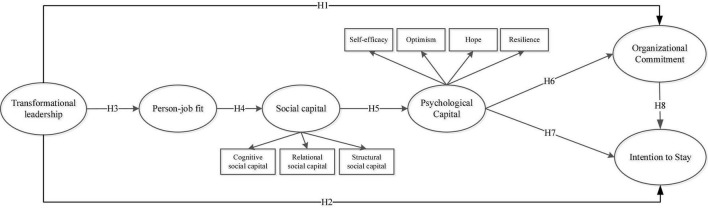
Research framework.

The theoretical framework of this study has been shown in Figure 1.

## Methodology

The data for this study were collected from performing arts organizations in China. This study concentrates on individuals associated with performing arts organizations. Hence, the data were collected from 15 top-ranking arts institutes located in Guangzhou City, China, and a total of 240 samples were collected using the convenience sampling technique. The data were collected using a questionnaire. A valid response rate of 93.33% and 224 respondents were received (Comrey and Lee, 1992; MacCallum et al., 1999). This questionnaire used a seven-point Likert scale. Hence, with the help of a Likert scale, researchers were able to get a more precise result by measuring how strongly an individual agreed or disagreed with a certain questionnaire item. This seven-point scale ranged from 1 (strongly disagree) to 7 (strongly agree), with four links to a neutral category. TL was calculated by items proposed by Manzoor et al. (2019), while the items to measure OC and SC were implemented from the study by Ko (2021). ITS was calculated by items proposed by Price (2001). Furthermore, the items to measure PJF were adopted from Islam et al. (2019). Finally, PC was measured by the items suggested by Luthans et al. (2007a). A pilot test was conducted prior to conducting the formal survey. The pilot test targeted 50 individuals associated with performing arts organizations. The test helped in testing and validating the questionnaire items. Partial least squares (PLS) were used to conduct the analysis of this research.

The PLS analysis was conducted in two stages. The first stage was related to conducting the reliability analysis, whereas the second stage was associated with testing the research framework of this study (Anderson and Gerbing, 1988; Hulland, 1999). PLS is considered to be a reliable tool in terms of calculating the validity and reliability of measurement items and analyzing the research framework (Petter et al., 2007). Moreover, PLS can evaluate variables with non-normal distributed data because of its certain features. Hence, it is considered to be a well-suited measurement tool in terms of assessing complicated research frameworks (Chin et al., 2003). Consequently, to assess the relationships among constructs, measure the errors, and avoid collinearity, PLS was deemed to be a much better option as compared with traditional SEM techniques.

## Data Analysis

### Convergent and Discriminant Validity

The Smart PLS version 3.2.8 was used to conduct the analysis of this study. Furthermore, the hypotheses of this research were analyzed employing the structural equation modeling (SEM) technique. To analyze complicated frameworks, researchers recommend the PLS-SEM method (Hair et al., 2014). In addition, PLS-SEM is well suited to conduct mediation analysis and calculate estimations, as compared to regression analysis (Preacher and Hayes, 2004). Similarly, the assumption related to validation of the normality is not mandatory while using PLS-SEM (Hair et al., 2014).

Both the outer and inner research models are included in PLS-SEM. The outer research framework’s reflective constructs were calculated using convergent validity and individual item reliability. The highest factor loading value of 0.957 and the lowest factor loading value of 0.789 are reported, as highlighted in Table 1. The factor loading values were above the threshold value of 0.7 and, hence, were considered acceptable (Hair et al., 2014). Consequently, it can be inferred that this research has satisfactory individual item reliability. The internal reliability of all the variables is calculated using composite reliability (CR). A threshold value greater than 0.6 is considered acceptable in terms of CR values (Hair et al., 2014). As indicated in Table 1, all the CR values are higher than 0.6; therefore, this study has an acceptable internal consistency (Bagozzi et al., 1991). The convergent validity of this study was analyzed, employing average variance extracted (AVE). The highest AVE value of 0.942 and the lowest AVE value of 0.686 are shown in Table 1. Consequently, this research has a satisfactory convergent validity because the AVE’s threshold value of 0.50 or more is considered to be acceptable (Hair et al., 2014).

**TABLE 1:** Convergent validity of constructs.

Construct	Cronbach’s alpha	Composite reliability	Average variance extracted (AVE)
TL	0.963	0.970	0.845
PJF	0.733	0.879	0.707
Cognitive SC	0.969	0.980	0.942
Relational SC	0.965	0.972	0.852
Structural SC	0.913	0.939	0.793
Hope	0.914	0.939	0.795
Optimism	0.864	0.936	0.880
Resilience	0.928	0.954	0.874
Self-efficacy	0.912	0.945	0.851
OC	0.938	0.953	0.803
ITS	0.948	0.963	0.865

*TL, transformational leadership; PJF, person–job fit; SC, social capital; OC, organizational commitment; ITS, intention to stay.*

Discriminant validity was employed to evaluate the differences between the constructs and their indicators. According to the results demonstrated in Table 2, the factor loading of all the indicators connected with its related construct exceeds the value of all other factor loading values in the latent structure. The highest factor loading values are highlighted in yellow in Table 2 (Hair et al., 2016).

**TABLE 2:** Standardized factor loadings and cross-loadings of the outer model.

Item/Construct	ITS	OC	PJF	PC	SC	TL
ITS1	0.938	0.742	0.610	0.599	0.631	0.670
ITS2	0.902	0.739	0.653	0.635	0.649	0.670
ITS3	0.950	0.763	0.584	0.606	0.616	0.630
ITS4	0.930	0.812	0.641	0.669	0.636	0.629
OC1	0.781	0.922	0.648	0.733	0.794	0.740
OC2	0.717	0.914	0.629	0.708	0.772	0.720
OC3	0.791	0.892	0.627	0.659	0.706	0.615
OC4	0.726	0.890	0.607	0.713	0.718	0.615
OC5	0.665	0.860	0.596	0.651	0.719	0.678
PJ1	0.651	0.645	0.866	0.656	0.559	0.491
PJ2	0.548	0.593	0.909	0.628	0.646	0.612
PC1	0.446	0.511	0.540	0.804	0.465	0.405
PC2	0.455	0.542	0.554	0.789	0.488	0.444
PC3	0.521	0.578	0.553	0.809	0.503	0.444
PC4	0.586	0.646	0.647	0.736	0.654	0.612
PC5	0.565	0.697	0.668	0.789	0.733	0.616
PC6	0.610	0.712	0.607	0.870	0.667	0.550
PC7	0.660	0.689	0.623	0.815	0.661	0.655
PC8	0.559	0.675	0.598	0.869	0.614	0.490
PC9	0.561	0.706	0.608	0.871	0.686	0.552
PC10	0.486	0.562	0.527	0.808	0.526	0.425
PC11	0.586	0.660	0.606	0.881	0.625	0.557
PC12	0.641	0.676	0.614	0.882	0.624	0.538
SC1	0.581	0.629	0.665	0.664	0.795	0.689
SC2	0.566	0.718	0.628	0.741	0.792	0.623
SC3	0.595	0.680	0.577	0.685	0.831	0.656
SC4	0.535	0.682	0.571	0.651	0.817	0.598
SC5	0.577	0.724	0.650	0.630	0.902	0.777
SC6	0.615	0.738	0.602	0.625	0.911	0.798
SC7	0.596	0.740	0.587	0.630	0.925	0.811
SC8	0.598	0.715	0.546	0.601	0.876	0.807
SC9	0.630	0.742	0.588	0.599	0.897	0.811
SC10	0.603	0.728	0.586	0.615	0.905	0.865
SC11	0.600	0.770	0.582	0.635	0.885	0.833
SC12	0.584	0.765	0.578	0.630	0.893	0.819
SC13	0.619	0.740	0.555	0.598	0.875	0.831
TL1	0.673	0.721	0.533	0.571	0.813	0.890
TL2	0.619	0.651	0.544	0.585	0.824	0.912
TL3	0.687	0.735	0.606	0.613	0.807	0.957
TL4	0.655	0.715	0.612	0.599	0.791	0.918
TL5	0.554	0.631	0.555	0.533	0.802	0.900
TL6	0.656	0.690	0.599	0.599	0.813	0.937

*TL, transformational leadership; PJF, Person–job fit; SC, social capital; PC, psychological capital; OC, organizational commitment; ITS, intention to stay.*

The goodness of fit (GOF) for this study was analyzed using the model proposed by Tenenhaus et al. (2005) to determine the quality of the proposed research model, which calculates as follows:


G⁢O⁢F=A⁢V⁢E¯⁢x⁢R2¯=0.837⁢x⁢ 0.743=0.789

Corresponding to the abovementioned calculation, the GOF is 0.789, achieving the cutoff condition (0.210) for a significant impact size (Wetzels et al., 2009).

### Empirical Results

**FIGURE 2:**
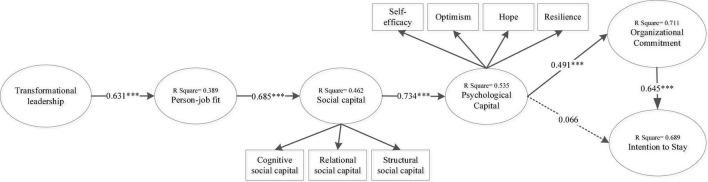
Research results. ^***^p < 0.001.

The inner model of this research is calculated by using Smart-PLS. The *p*-values and *t*-values are analyzed to investigate the proposed hypotheses in the inner model. The suggested hypotheses are supported when *p* < 0.05 or *t* > 1.96. According to the results of this research, as shown in Table 3 and Figure 2, TL was discovered to have a positive impact on OC (β = 0.444, *t* = 5.268) and PJF (β = 0.631, *t* = 9.796), hence, supporting H1 and H3, while having no significant effect on ITS (β = 0.171, *t* = 1.571), thus rejecting H2. Furthermore, PJF had a significant impact on SC (β = 0.685, *t* = 12.621); therefore, H4 was supported. Moreover, SC significantly influenced PC (β = 0.734, *t* = 17.536); therefore, H5 was supported. PC was discovered to be in a significant correlation with OC (β = 0.491, *t* = 6.693), hence, supporting H6, while having no significant association with ITS (β = 0.066, *t* = 0.876), consequently rejecting H7. Finally, OC was also in a significant relationship with ITS (β = 0.645, *t* = 5.099), indicating H8 was supported.

**TABLE 3:** Hypothesis results.

Hypothesis	Path coefficient (β)	*T* statistics	*P-*values
H1: TL - > OC	0.444	5.268	0.000
H2: TL - > ITS	0.171	1.571	0.116
H3: TL - > PJF	0.631	9.796	0.000
H4: PJF - > SC	0.685	12.621	0.000
H5: SC - > PC	0.734	17.536	0.000
H6: PC - > OC	0.491	6.693	0.000
H7: PC - > ITS	0.066	0.876	0.381
H8: OC - > ITS	0.645	5.099	0.000

*TL, transformational leadership; PJF, person–job fit; SC, social capital; PC, psychological capital; OC, organizational commitment; ITS, Intention to stay.*

Figure 2 also indicates the values of R^2^. These values represent the coefficient of determination values for exogenous variables. Corresponding to the results, OC possesses a value greater than 0.7, which is deemed as a strong effect size. In contrast, PC and ITS possess values between 0.5 and 0.7 and, hence, are considered to have a moderate effect. Finally, PJF and SC indicate a weak effect because their values lie in between the values of 0.3 and 0.5 (Moore et al., 2013).

According to the results shown in Table 4, TL had an indirect significant relationship with SC (β = 0.435, *t* = 5.864) and PC (β = 0.321, *t* = 4.808). Moreover, TL was also found to have a significant indirect relationship with OC (β = 0.217, *t* = 4.068) and ITS (β = 0.098, *t* = 2.954).

**TABLE 4:** Indirect effects.

Indirect path	Path coefficient (β)	*T*-values	*P*-values
TL- > PJF- > SC	0.435	5.864	0.000
TL- > PJF- > SC- > PC	0.321	4.808	0.000
TL - > PJF - > SC - > PC - > OC	0.217	4.068	0.000
TL - > PJF - > SC - > PC - > OC > ITS	0.098	2.954	0.003

*TL, transformational leadership; PJF, person–job fit; SC, social capital; PC, psychological capital; OC, organizational commitment; ITS, intention to stay.*

## Discussion

### Conclusion

A significant methodology is required to tackle the complicated concepts of TL and social factors in an arts industry context. This article aims to observe the direct impacts of TL on OC. Furthermore, it examined the indirect effects of TL on OC and ITS *via* PJF, SC, and PC. This article creates a conceptual model based on fit theory, SC theory, and theory of PC by Avey et al. (2009). According to this study, TL was in a significant relationship with OC. These results were in accordance with a study conducted by Rego et al. (2016). However, according to the findings of Rego et al. (2016), only three of the four dimensions of PC (i.e., self-efficacy, hope, and optimism) significantly impacted OC. Moreover, corresponding to the results of this study, TL was also found to be in a significant relationship with PJF. These findings are somewhat similar to the study by Bui et al. (2017). Their research demonstrated that TL significantly impacted work engagement, and this association was mediated by PJF in a Chinese context (Bui et al., 2017). However, TL was found to be in an insignificant association with ITS. These results were different from the previous literature. According to previous research, TL had a positive and significant impact on ITS (Sobaih et al., 2020).

Furthermore, this research indicated a significant relationship between PJF and SC. This result was similar to a research study that explored TL, SC, and PJF. In support of the hypotheses, TL was positively related to both PJF and SC. In addition, PJF was a significant mediator for the relationship between TL and SC (Raja et al., 2018). Moreover, SC was also found to be in a significant relationship with PC. The result was in accordance with a study that analyzed SC, PC, and entrepreneurial intent. According to the findings, SC was significantly associated with PC (Mahfud et al., 2020). In addition, PC was significantly associated with OC. The results were similar to research conducted on nurses in the Xi’an City of China. The study reported the influence of PC on job burnout by examining the mediating impact of OC on this association. The conclusions showed a significant relationship between PC and OC (Peng et al., 2013). However, according to this study, PC did not significantly impact on ITS. The results are dissimilar to a study using conservation of resources (CORs) and congruence theories as to their theoretical background and analyzed a framework, which explored the impact of PC on family–work conflict, work–family conflict, absence of intentions, and ITS. According to their results, PC was significantly influencing employees’ turnover intention (Karatepe and Karadas, 2014). Finally, corresponding to the results of this study, OC significantly influenced ITS, and these results were somewhat similar to a study conducted by Ibrahim Alzamel et al. (2020).

This study also analyzed the indirect effects of TL with OC and ITS. According to the results of the study, TL had a significant indirect relationship with OC. This finding is somewhat similar to a study conducted by Rego et al. (2016). According to the results of their study, TL was in a significant indirect relationship with OC with PC as a mediator. Furthermore, according to the results of this research, TL was found to be a significant indirect relationship with ITS. This result is somewhat similar to a study that measured the indirect relationship of TL with ITS. The results of the aforementioned study indicated a significant indirect relationship with ITS with OC as a mediator (Sobaih et al., 2020).

### Theoretical Implications

This study theoretically adds to the expanding body of investigation on TL by analyzing its direct and indirect impacts on two important concepts in HRM that are OC and ITS. Moreover, it theoretically improves the earlier studies related to the fit theory, SC theory, and theory of PC by Luthans et al. (2007b) in the context of the art industry. This research similarly provides empirical support, proving that the significant correlation between TL and PJF is faced in non-western environments. Simultaneously with the discoveries by Nguyen et al. (2017), this research combines further proof of optimistic effects of TL in non-western societies. This study demonstrates that TL is a significant factor as PJF performs a significant role in workers’ SC. It improves our knowledge of workers’ SC further, suggesting that SC is affected by fit theory, which concentrates on worker fit to the job as an antecedent of SC. This research adds to the fit theory by explaining that workers’ SC is significantly affected by their assessments on PJF that can be enhanced by TL.

Creative employees in performing arts industries need flexibility and independence to work in a creative production environment. Nevertheless, the organizational capital of various industries comprises formal rules, hierarchies, and organizational structures that might hinder the creativity of employees working in performing arts industries, which in turn decreases the OC of employees. Hence, in the case of managing the employees of performing arts industries, companies should provide an environment that promotes and encourages creativity. In performing arts industries, SC increases OC through PC; hence, investments in SC might result in increases in employees’ skills and capabilities. Consequently, creative employees are deemed to have a positive work attitude because of the organizational investments in SC, which result in high OC (Chew and Chan, 2008).

The SC highlights social communication in external relations of companies. These networks enable knowledge exchange and information creation. Creative employees will cooperate and trade concepts with their associates. Furthermore, they will collect information by solving several challenges with the help of network associations. In addition, resources acquired through networks offer socio-emotive assistance for people (Ibarra, 1993), and these optimistic personal relations will improve trust, collaboration, and understanding among the employees of performing arts industries. Corresponding to the advantages taken from SC, creative employees will have higher OC (Chen et al., 2012).

### Practical Implications

The study will make the following practical implications to the performing arts organizations. First, performers often have a unique with persistent understanding of the arts, and they are less sensitive to rules and are not satisfied with passive obedience in their work. Performers often have a strong sense of professional identity, but their passion for the art itself is not enough to promote their desire to remain with a performing arts organization (Brodsky, 2006). Therefore, managers of performing arts organizations can make considerable efforts to increase communication between the organization and the performers to better learn their employees’ work positive and adverse conducts. Second, arts organizations are recommended to concentrate more on investments in performers’ careers, with the help of offering them future learning prospects and opportunities in order to enhance their creative abilities. This investment in human capital will enhance the OC of employees. Third, managers of arts industries should create various interaction networks, place physical settings that assist individual communications, and reward activities of information distribution (Chen et al., 2012). Fourth, managers are advised to encourage OC in their organizations by promoting the philosophy of mutual communication with their employees (Afsar et al., 2018). One way of enhancing performers’ commitment to their organization is to increase their autonomy as decision-makers (Oakland and Ginsborg, 2014). Transformational leaders value the development of their employees and increase their participation in decision-making. Therefore, it is recommended that leaders of performing arts organizations adopt a transformational approach to increase performers’ OC. Finally, this research article offers detailed and comprehensive guidelines for managers to adopt an effective leadership strategy and engage with their employees to ensure smooth operations in the organization. Hence, it is recommended for managers and practitioners to adopt TL because of its ability to successfully enhance employees’ OC and ITS (Sobaih et al., 2020).

### Limitations and Future Research

This article has some limitations but also provides a direction for future research. The research objective of this study is the leadership of performing arts organizations impacted by societal norms in cultural industries. The creation and implementation of creative concepts are motivated by the personal psychological actions of individuals, and capital is created through leadership. Nevertheless, whether the findings of this research can be employed in other industries, there are still other industries that can be explored using this research framework. Furthermore, this study was conducted in the context of China’s performing arts industry, which is influenced by Confucian culture, where individuals tend to follow the behavior of the masses. Social norms may have different influences on creativity and leadership in different cultural contexts. Therefore, future research can start from the background of social norms in different countries to explore the social factors that enhance creativity and leadership. In addition, this study used a survey as a research methodology. Different individuals have distinct interpretations of similar items; consequently, it can be indicated that the questionnaire findings might have some impact on the ultimate empirical outcomes. The questions applied in the survey are close-ended, which has the possibility of omitting some additional comprehensive and deep knowledge. Therefore, future researchers can employ case study methodology in combination with survey research to get a more detailed analysis.

Finally, future researchers can modify the constructs used in this study. For instance, this research used PC as a second-order construct and included all four dimensions of PC, namely, self-efficacy, hope, resilience, and optimism. Hence, considering Luthans et al. (2007a) previous research argument about investing in all the dimensions of PC as a whole into account, future researchers are recommended to conduct a study in which they can measure the individuals’ leadership capability by conducting a comparison between two groups. One group can be measured by taking into account only some dimensions of PC, while the other group can be measured by taking all the four dimensions of PC into account. This analysis cannot only provide proof for the theories of researchers, such as Luthans et al. (2007b), but also demonstrate the importance of using some dimensions of PC, as conducted previously by some researchers (Gardner et al., 2005; Walumbwa et al., 2008; Rego et al., 2016).

## Data Availability Statement

The raw data supporting the conclusions of this article will be made available by the authors, without undue reservation.

## Author Contributions

HX, ZW, and AK: conceptualization and methodology. HX: formal analysis, investigation, and visualization. HX, ZW, NL, AK, and LZ: writing about original draft preparation, and review and editing. All authors have agreed to the published version of the manuscript.

## Conflict of Interest

The authors declare that the research was conducted in the absence of any commercial or financial relationships that could be construed as a potential conflict of interest.

## Publisher’s Note

All claims expressed in this article are solely those of the authors and do not necessarily represent those of their affiliated organizations, or those of the publisher, the editors and the reviewers. Any product that may be evaluated in this article, or claim that may be made by its manufacturer, is not guaranteed or endorsed by the publisher.
